# The Role of Chloride ion in the Silicate Condensation Reaction from ab Initio Molecular Dynamics Simulations

**DOI:** 10.1021/acs.jpcb.3c04256

**Published:** 2023-08-30

**Authors:** Thi H. Ho, Tuong Ha Do, Hien Duy Tong, Evert Jan Meijer, Thuat T. Trinh

**Affiliations:** † Laboratory for Computational Physics Institute for Computational Science and Artificial Intelligence, Van Lang University, Ho Chi Minh City 700000, Vietnam; ‡ Faculty of Mechanical - Electrical and Computer Engineering School of Technology, Van Lang University, Ho Chi Minh City 700000, Vietnam; ¶ Faculty of Applied Sciences, Ton Duc Thang University, 19 Nguyen Huu Tho, Tan Phong ward District 7, Ho Chi Minh City 700000, Vietnam; § Faculty of Engineering, Vietnamese-German University (VGU), Thu Dau Mot City, Binh Duong Province 75000, Vietnam; ∥ Van ’t Hoff Institute for Molecular Sciences, University of Amsterdam, Amsterdam 1012 WX, The Netherlands; ⊥ Porelab, Department of Chemistry, Norwegian University of Science and Technology, NO-7491 Trondheim, Norway

## Abstract

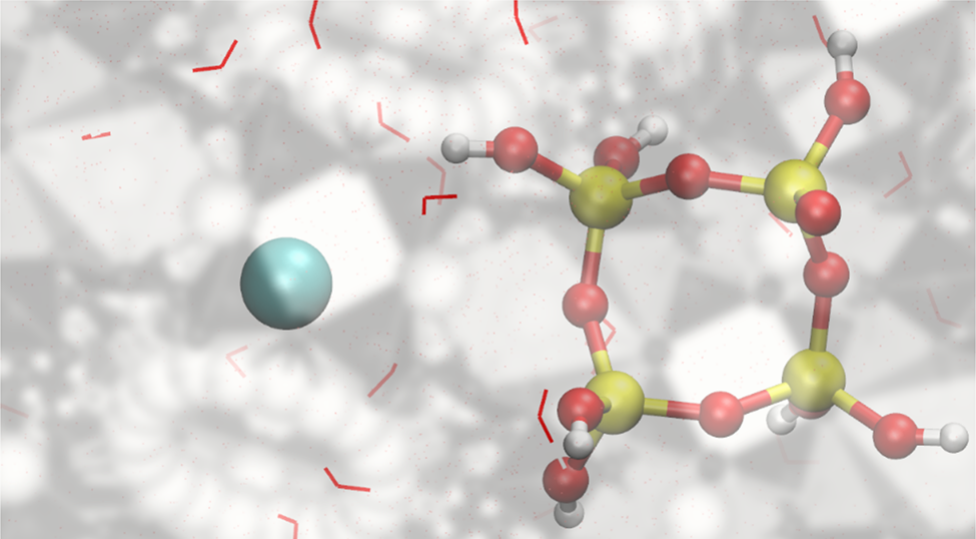

The comprehension of silicate oligomer formation during the initial stage of zeolite synthesis is of significant importance. In this study, we investigated the effect of chloride ions (Cl^–^) on silicate oligomerization using *ab initio* molecular dynamics simulations with explicit water molecules. The results show that the presence of Cl^–^ increases the free energy barriers of all reactions compared to the case without the anion. The formation of the 4-ring structure has the lowest free energy barrier (73 kJ/mol), while the formation of the 3-ring structure has the highest barrier (98 kJ/mol) in the presence of Cl^–^. These findings suggest that Cl^–^ suppresses the formation of 3-rings and favors the formation of larger oligomers in the process of zeolite synthesis. Our study provides important insights into the directing role of Cl^–^ in silicate oligomerization by regulating thermodynamic and kinetic parameters. An important point to consider is the impact of the anion on aqueous reactions, particularly in altering the hydrogen bond network around reactive species. These results also provide a basis for further studies of the formations of larger silicate oligomers in solution.

## Introduction

Zeolites are aluminosilicate materials with nanoporous structures that exhibit excellent catalytic and separation properties, making them widely used in various industrial applications.^1^ The synthesis of zeolites typically involves the use of aqueous gel solutions containing various heteroatomic compounds, with inorganic or organic cations acting as directing agents of the structures. The nature and structure of the silicate oligomers in solution have been extensively studied experimentally, as understanding the formation of silicate oligomers in the initial stage is key to zeolite synthesis.^2−10^ Computational studies^11−21^ using a continuum or explicit model of water^22−26^ have also been conducted to investigate the initial steps of silicate oligomerization. A common pathway of the oligomerization reaction is a two-step mechanism with an initial formation of a pentacoordinated intermediate, followed by a water removal stage. Earlier studies^26−28^ have shown that it is crucial to include the effect of thermal motion and the presence of explicit water molecules when modeling aqueous chemical reactions that involve solvent molecules that strongly bind to the reagents or actively participate in the reaction mechanism. The impacts of the charged ion have an important role in the silicate condensation reaction. For example, the organic cations (such as tetramethylamine (TMA^+^), tetraethylamine (TEA^+^), tetrapropyl amine (TPA^+^)) were shown to have a decisive role in the formation of dominating silicate spices during the initial state of zeolite formation.^29,30^ A detailed picture of the interaction of inorganic cations (such as Li^+^, Na^+^, ) was also reported to have great impacts on the activation barrier of the condensation reaction.

In contrast to extensive research on the effect of cations on silicate reactions, the role of anions in this process has received relatively little attention. Recent work by Do et al.^31^ has demonstrated that OH^–^ ion play a critical role in silicate condensation reactions by directly participating in these reactions. Chloride ion is another ubiquitous and crucial ion in various chemical reactions. Despite its known significance, the role of the chloride ion in zeolite synthesis has not been fully explored. Liu et al.^32^ investigated the impact of anions on the synthesis of NaA zeolite using the dry-gel conversion method. The authors explored the role of the alkalinity of the reaction system and two aluminum sources, aluminum chloride and aluminum sulfate, in the crystallization process. They observed that the anions have a pronounced effect on the synthesis of NaA zeolite, which can be attributed to their electrostatic and steric interactions with the zeolite framework.^32^ Therefore, understanding the effect of chloride ion on the formation of silicate oligomers and its subsequent impact on the zeolite structure can provide valuable insights into the mechanism of zeolite synthesis, ultimately leading to improved control over the synthesis process and the development of new and more efficient synthesis strategies.

In this study, the formation of silicate oligomers in the presence of Cl^–^ in aqueous solution was investigated by using *ab initio* molecular dynamic (AIMD) simulations, which explicitly included the water molecules. The study aimed to understand the role of Cl^–^ in the formation of different silicate species, ranging from dimer to 4-ring, which are crucial intermediates in the initial stage of zeolite synthesis. The free energy profiles of the formation pathways of these silicate oligomers were obtained by calculating the activation energy required for each step of the reaction. The simulations revealed that the presence of Cl^–^ has a significant impact on the activation energy required for the silicate condensation reaction. The calculated free energy profiles of the reaction pathways showed that the formation of a 4-ring structure is energetically favored over the formation of a 3-ring structure in the presence of Cl^–^. The results of this study provide insights into the role of Cl^–^ in controlling the formation of different silicate species during the initial stage of zeolite synthesis. The findings could potentially aid in the design of new synthetic routes for the controlled synthesis of zeolites with the desired properties.

The fluoride route has been considered as one of the alternative paths in zeolite synthesis.^1,33^ However, the preference for the fluoride route stems from the potential differences it might introduce in the mechanism of silicate condensation reactions.^33^ In our attempt to unravel the influence of anions in zeolite synthesis conditions, we have chosen to focus on the chloride ion, marking an initial effort in understanding the role of anions in this context. The chloride ion might be deemed less intricate than its counterpart, the fluoride ion. However, the results show a surprising insight: that the chloride ion, too, plays a pivotal role in governing the silicate condensation reactions.

## Simulation Method

Our study employed a computational setup that is similar to earlier studies investigating silicate oligomerization reactions in aqueous solution.^23,24,27^ Specifically, we performed AIMD simulations based on a density functional theory (DFT) approach to describe the electronic structure. The simulations were carried out using the CP2K package,^34^ and the Born–Oppenheimer approach was implemented in the Quickstep module.^35^ To account for the interactions between the core electrons and atomic nuclei, we used the Goedecker–Teter–Hutter (GTH) pseudopotentials.^36,37^ The BLYP exchange-correlation functional^38,39^ was used, together with Grimme’s type D2,^40^ to account for long-range van der Waals interactions. The double-ζ valence (DZVP-MOLOPT) basis set with polarization functions^41^ was employed for all atom types, and an energy cutoff of 400 Ry was chosen for the auxiliary plane wave basis set.

To generate molecular dynamics trajectories, we used a time step of 0.5 fs. To impose a temperature of 350 K, we applied a velocity rescaling thermostat^42^ with a time constant of 300 fs. Our simulation approach allowed us to obtain free energy profiles of the formation pathways of different silicate oligomers in the presence of chloride ions. The simulation cell was a periodic cubic box (16 × 16 × 16 Å^3^) with a density around 1 g/cm^3^, similar to that of the experimental values. The initial geometry of the silicate oligomer and Cl^–^ ion was first optimized in the gas phase. This structure was then solvated with around 128 water molecules evenly distributed in the simulation box. No cations were added to the system. Instead, a positive neutralizing background charge was imposed to balance the system’s negative charge.^34^ Subsequently, to generate a representative configuration, a 20 ps equilibration run was performed in the NVT ensemble. The total number of atoms in the system was approximately 450 atoms. Due to the high simulation cost of *ab initio* MD, we did not consider systems with a higher concentration of silicate and Cl^–^ in the present study.

Reaction pathways were obtained by tracing a proper reaction coordinate using the method of constraints.^43,44^ For each value of the reaction coordinate, the initial configuration was taken from the last configuration of the simulation at the previous value of the reaction coordinate. After 1 ps of equilibration, a 10 ps trajectory was generated to gather data. The total trajectory of the simulations of a reaction pathway was around 200 ps, distributed typically over 20 values of the reaction coordinate.

**Scheme 1:**
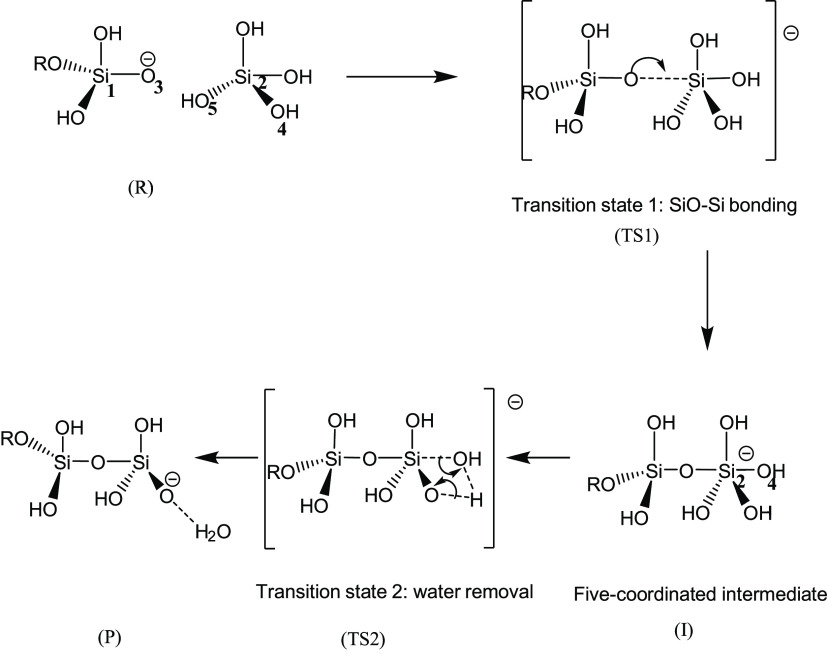
Representation of a Two-Step Mechanism of Silicate Condensation Reaction with the Presence of Cl^–^ R, TS1, I, TS2, and P refer to reactant, transition state 1, intermediate, transition state 2, and product, respectively.

The free energy (Δ*G*) profiles of the oligomerization reactions were obtained by numerical integration using eq 1
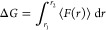
1Here, *r* denotes the reaction coordinate, and *F* is the calculated constraint force at a fixed value of the reaction coordinate. As illustrated in Scheme 1, the reaction coordinate *r* is typically the bonding distance between O_3_–Si_2_ and Si_2_–O_4_ for the first and second steps of the silicate condensation reaction, respectively. *r*_1_ denotes the value of the reactant state, and *r*_2_, that of the product state. The intergral is evaluated numerically on the basis of the calculated values of the constraint force at each of the reaction coordinate values. The errors of the constraint force are typically below 10^–5^ Hartree/Bohr in a 10 ps production run. This approach has generic applicability and is used extensively in earlier studies to calculate free energy barrier reactions in solution.^23,45−48^

It is known that at basic conditions, the equilibrium between silicic acid Si(OH)_4_ and its deprotonated form Si(OH)_3_O^–^ occurs via the reaction 2
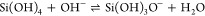
2

After the deprotonation step of silicic acid, the first silicate condensation reaction begins as described in reaction 3. Subsequent silicate condensation reactions, as described in reactions 4–7, lead to the formation of higher oligomers and ring structures.
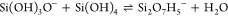
3
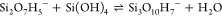
4
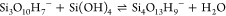
5
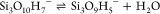
6
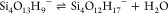
7

**Figure 1:**
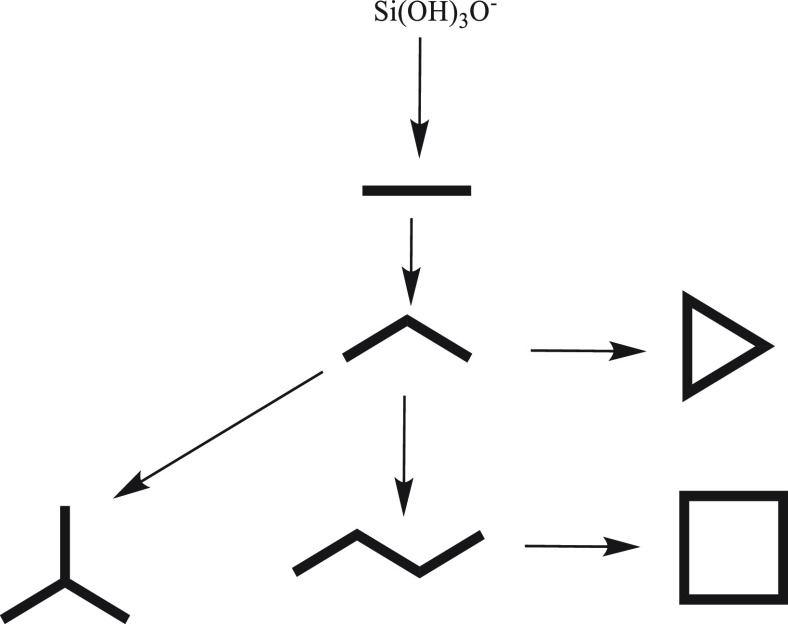
Scheme of the silicate oligomerization reactions considered in this work forming from dimer to 4-ring formation. All species are negatively charged, as described in Scheme 1.

A common two-step mechanism of silicate condensation reaction in the basic conditions^27^ is described in Scheme 1. The first step is to form a 5-fold coordinated intermediate with OSi–O bonding. For this stage of the reaction pathway, the distance between atoms of O_3_ and Si_2_ was taken as the reaction coordinate. Here, the O_3_ atom is defined as the reactive oxygen. The second stage consists of a water removal step, where the distance between Si_2_ and O_4_ was taken as the reaction coordinate. For ring closure reactions, a similar mechanism has been considered.^27,49^ Note that in the ring closure reaction, the silicon and oxygen atom in the first reaction step are of the same oligomer molecule. We investigated 6 oligomerization reactions, from the formation of a dimer up to a 4-ring structures. A schematic process of the reactions is provided in Figure 1. For each reaction, *ab initio* MD with an explicit water model was used to calculate the free energy profile and elucidate the reaction mechanism.

## Results and Discussion

### Radial Distribution Functions

**Figure 2:**
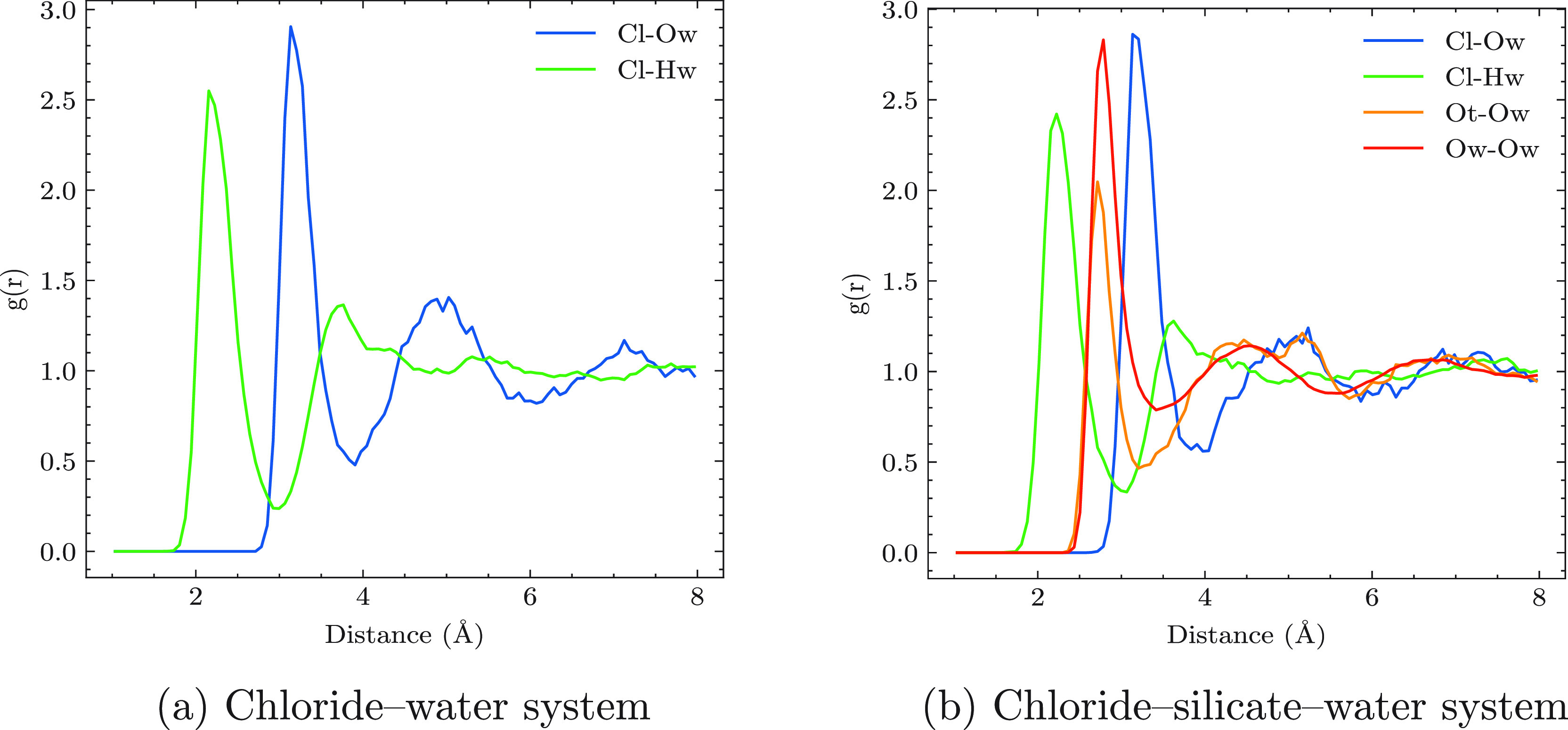
Radial distribution function of different atom pairs for a Cl^–^ solvated in water (a) and Cl^–^ – silicate solvated in water (b), as obtained by unconstrained *ab initio* MD simulations. See the text for more details.

The radial distribution functions (RDFs) of chloride–oxygen (Cl-Ow) and chloride–hydrogen atoms (Cl-Hw) of water are crucial to understanding the structure and behavior of these species in aqueous solution. In this section, we present the RDFs of Cl-Ow and Cl-Hw obtained from an unconstrained 20 ps AIMD simulation of two independent systems: Cl^–^–water and Cl^–^–Si(OH)_3_O^–^–water systems. The RDF plot of the chloride-water, depicted in Figure 2a, exhibits the expected peaks, with the first peak indicating the nearest-neighbor of water around the chloride ion, while the first mininum in RDF incidates the position of the first solvation shell. The calculated coordination numbers of Cl-Ow and Cl-Hw are found to be 6.3 and 5.7, respectively. These results are in good agreement with previously reported data from neutron diffraction^50^ and X-ray scattering^51^ experiments as well as simulation data^52^ (see Table 1).

**Table 1:** Calculated Values Obtained from AIMD Simulations of the Position of the First Minimum in RDFs and the Corresponding Coordination Numbers for Cl-Ow and Cl-Hw^a^

Method	*r*_Cl–Ow_^*min*^ (Å)	*n*_Cl-Ow_	*r*_Cl–Hw_^*min*^ (Å)	*n*_Cl-Hw_
BLYP-vdW (this work)	3.87	6.3	2.97	5.7
PBE-vdW (ref (52))	3.78	6.3	2.93	5.2
PBE0-vdW (ref (52))	3.73	6.3	2.9	5.5
Exp. (ref (50))		6.9 ± 1.0		6.0 ± 1.1
Exp. (ref (51))		6.4 ± 1.0		

a The values from the literature are added for comparison.

The structural characteristics of the solvation shell surrounding silicate and chloride ions in aqueous solution were also investigated. Figure 2b shows the effect of chloride and silicate monomer on the RDF of water. The first peak of the Ow-Ow RDF is located at 2.85 Å, which is consistent with experimental data and earlier simulations.^53^ This suggests that the presence of Cl^–^ and anionic silicate has no significant effect on the structures of water molecules. Taking the first minimun of the RDF in Ot-Ow and Cl-Ow as the position of the first solvation shell, the results show that the structure of the first solvation shell of the silicate-water and chloride-water is located at 3.25 and 3.87 Å, respectively (see Figure 2b). Interestingly, when anion silicate monomer and chloride ions are combined in solution, their solvation shells exhibit a closer contact than that observed in the individual systems. Specifically, the average of minimum distance between the chloride ion and oxygen atoms of silicate (Cl-Ot) is approximately 5 Å, which is shorter than the sum of the respective first solvation shell radii in the separate systems (6.0 Å). These findings suggest that the presence of both silicate and chloride ions may induce a change in the solvation structure of each ion, leading to an increased proximity between them.

### Formation of Linear Silicate Oligomers

**Figure 3:**
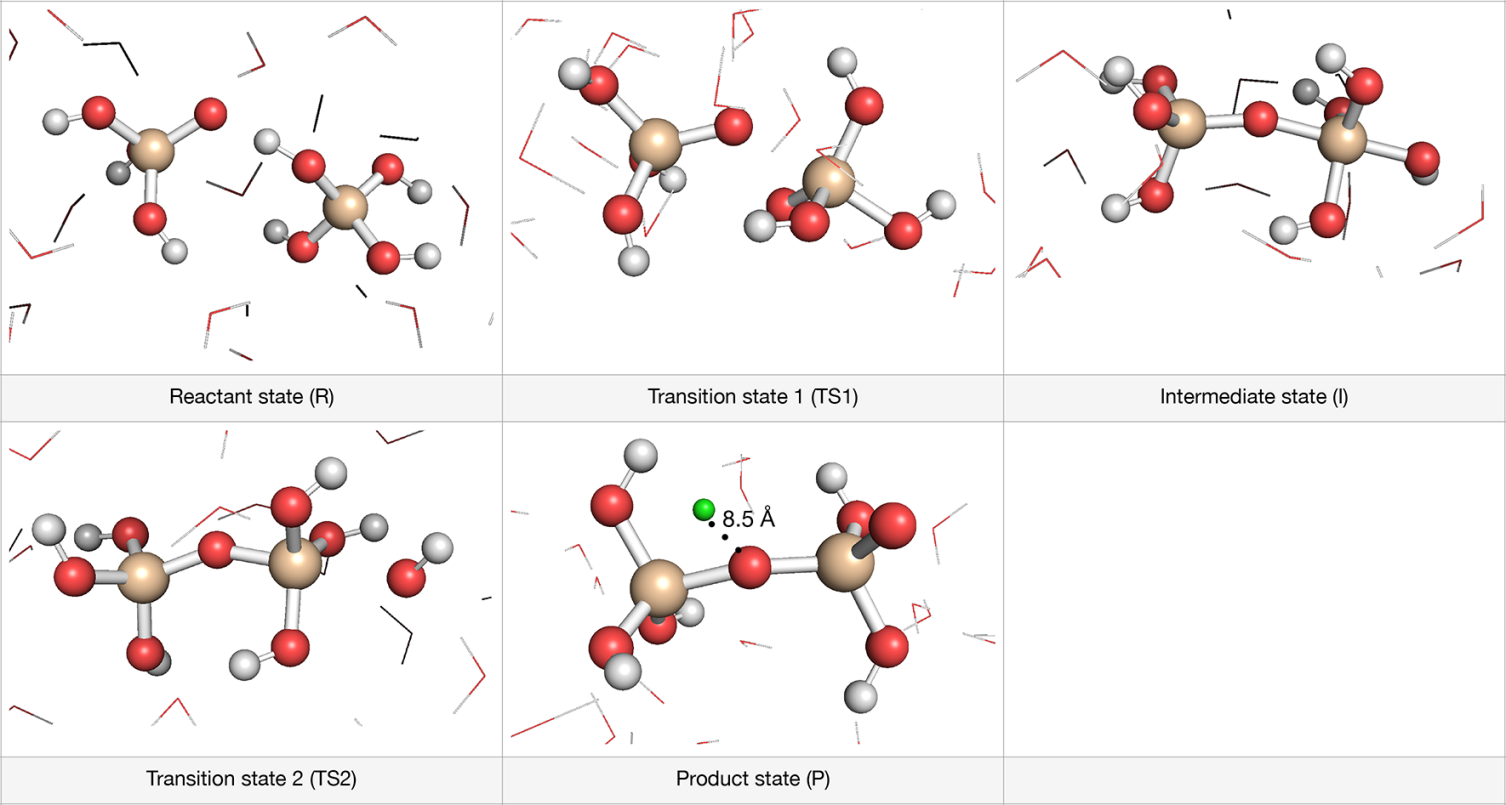
Representative snapshot from *ab initio* MD simulations of the dimerization reaction with the mechanism following in Scheme 1. The anion Cl^–^ stays far away from the silicate structure along the whole reaction pathway. In the product state, the shortest distance between Cl^–^ and silicate is 8.5 Å. The Si, O, H, and Cl atoms are colored yellow, red, white, and green, respectively. The water solvent is shown by using a stick model.

A representative snapshot obtained from AIMD simulations of the dimerization reaction following the mechanism presented in Scheme 1 is shown in Figure 3. Throughout the reaction pathway, the negatively charged chloride anion Cl^–^ is observed to be located far from the silicate structure.

The reaction mechanism for silicate condensation is consistent with previous studies.^27,49^ As shown in Scheme 1, the first reaction step involves the formation of a SiO–Si bond, resulting in a 5-fold silicate intermediate. The second step involves the removal of water molecules to produce the dimer product. Table 2 lists the calculated free energies of the transition states, intermediates, and products. Using the reactant as a reference, the free energy value of the first step of the dimerization reaction barrier was found to be 62 kJ/mol. The second activation barrier, which corresponds to the removal of water to form the dimer product, was calculated as the free energy difference between the transition state TS2 and the intermediate and was found to be 34 kJ/mol. The resulting overall activation barrier of dimer formation was found to be 81 kJ/mol. These values indicate that the dimerization reaction is energetically unfavorable. The calculated free energies of the transition states, intermediates, and products for the silicate dimerization reaction in the presence of chloride ions are listed in Table 2.

**Table 2:** Calculated Free Energy (kJ/mol) Profiles along the Silicate Formation in the Presence of Cl^–^ Ions Obtained by AIMD

Free energy	reactant	TS1	intermediate	TS2	product
Dimer	0	62	47	81	16
Trimer	0	59	48	76	22
Linear tetramer	0	64	48	77	23
3-ring	0	72	60	98	45
4-ring	0	63	49	73	6
Branched tetramer	0	60	45	77	31

It is well-established in the literature^24,29,49^ that the presence of ions can significantly influence the reaction kinetics and energetics. In this study, the obtained results indicate that the presence of Cl^–^ has a noticeable effect on the overall activation barrier of the dimerization reaction. Specifically, the presence of Cl^–^ increases the total activation barrier of the reaction by approximately 20 kJ/mol, when compared with the case without an ion (see Table 3). This result is consistent with previous reports in the literature, which suggest that counterions can significantly impact the reaction energetics by altering the charge distribution and the solvation environment of the reaction intermediates.^24^ Furthermore, it was found that the effect of Cl^–^ on the reaction kinetics is similar to that of the commonly used structure-directing agent, TMA^+^, which increases the overall activation barrier of the reaction by approximately 15 kJ/mol.^49^

**Table 3:** Total Free Energy Barriers (kJ/mol) Obtained by *ab Initio* MD of Silicate Oligomerization Reaction with the Presence of Cl^–^a

Free energy barrier	With Cl^–^ AIMD (this work)	Without Cl^–^ AIMD ref (23)	Neutral DFT ref (17)	Anionic DFT ref (55)
Dimer	81	61	193	66
Trimer	76	53	192	69
Linear Tetramer	77	/	/	53
3-ring	98	72	109	88
4-ring	73	95	111	61
Branched Tetramer	77	101	/	/

a The energies in the absence of Cl^–^ obtained from previous computaional works with AIMD^23^ and DFT^17,55^ approaches are added for comparison.

Upon analyzing the free energy barriers of the second step in the formation of dimer, trimer, and linear tetramer, it is found that these values range from 24 to 38 kJ/mol. These values and the reaction mechanism are in good agreement with previous studies of systems with different cations, such as Li^+^, ,^24^ and Na^+^.^27^ In the second step of silicate condensation, the leaving hydroxyl group forms hydrogen bonds with water molecules, which are essential for the water removal reaction.^49,54^ The protonation of the leaving hydroxyl group can occur via two pathways: direct protonation by another silicate hydroxyl group or a proton transfer chain mediated by one or more water molecules. The proton transfer chain is facilitated by the formation of a well-defined hydrogen bonding network between silicate and water, which enables efficient proton transfer.^54^ These observations are in agreement with previous studies, which have shown that the hydrogen bonding network plays a crucial role in determining the reaction mechanism of silicate condensation.^54^

Earlier studies have highlighted that the direct interaction between counterion and the active oxygen in the first step leads to a significant increase in the first activation barrier and, consequently, the overall barrier.^24,27^ In contrast, in this study, analysis of the trajectories involved in the formation of dimer, trimer, and linear tetramer revealed that the active oxygen in the first step (O_3_ in Scheme 1) does not have any direct contact with the chloride ion due to electrostatic repulsion. Instead, the chloride ion has a noticeable effect on the activation barrier due to the alteration in the first solvation shell of the silicate. The modification of the solvation shell by the presence of chloride ion likely leads to changes in the hydrogen bonding network between the silicate and water, which ultimately affects the activation barrier for the formation of the silicate dimer as discussed in the previous section.

**Figure 4:**
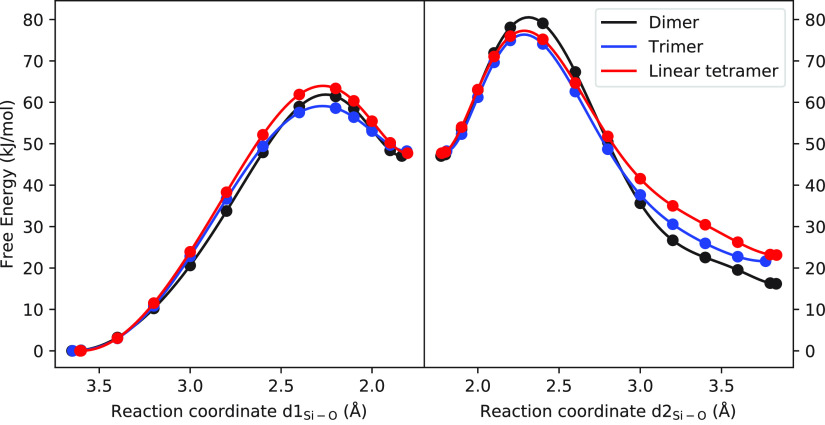
Calculated free energy profile (kJ/mol) of formation of linear silicate oligomer as a function of reaction coordinate.

By comparing the free energy values for the formation of linear species such as dimer, trimer, and tetramer, as presented in Table 3 and in Figure 4, it is evident that the activation energy is highest for the dimer and lower for the trimer and linear tetramer. The relative stability of the dimer product seems to be greater than those of the trimer and linear tetramer, indicating that the rate-limiting step for the linear growth of silicate in the presence of Cl^–^ is the formation of the dimer. This trend is in contrast to the case of TMA^+^, where the formation of a linear tetramer has the lowest overall activation barrier among the linear species.^49^ The lower activation energy for the trimer and linear tetramer suggests that the growth beyond dimerization occurs more rapidly than the initial step.

We also compared our findings on silicate condensation with previous studies conducted on the same subject in the presence of inorganic cations.^24,27^ Our observations suggest that the relative barrier heights for dimer and trimer formation in the presence of Cl^–^ are similar to those observed in the presence of the  cation, where the barrier for dimer formation is higher than that for trimer formation. In contrast, in the presence of Na^+^, the barriers for dimer and trimer formation are comparable, whereas the Li^+^ cation has the highest barrier for the trimer. These results indicate that the dominant linear species involved in silicate growth significantly differs with the presence of inorganic cations and anions.

### Formation of Ring and Branched Silicate Oligomers

**Figure 5:**
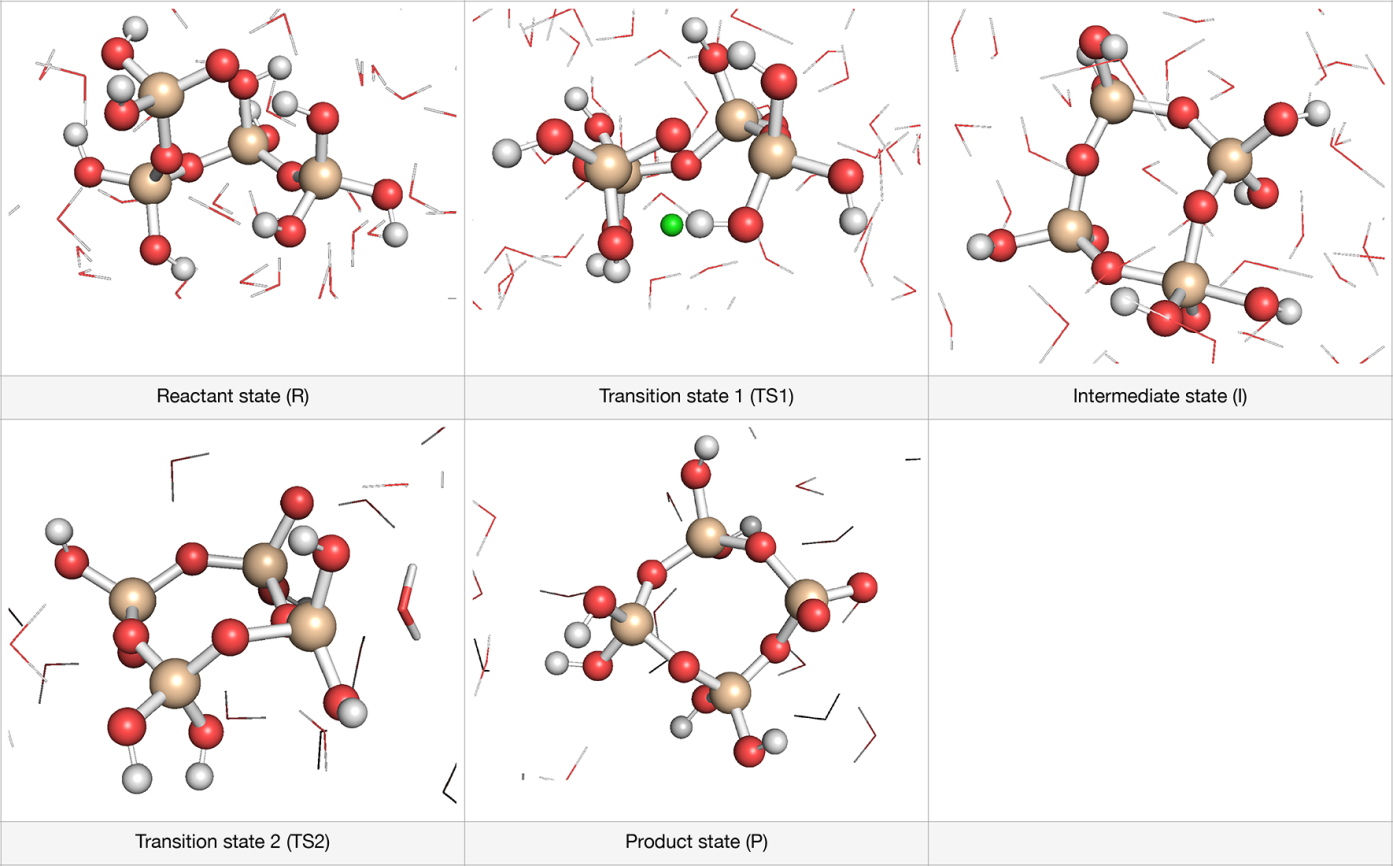
Representative snapshot from *ab initio* MD simulations of the 4-ring formation. The anion Cl^–^ stays far away from the silicate structure along the whole reaction pathway. The Si, O, H, and Cl atoms are colored yellow, red, white, and green, respectively. The water solvent is shown using a stick model.

The synthesis of zeolites involves the critical step of forming branched or ring structures at the initial stage of silicate production. The creation of initial 3-ring or 4-ring structures is a crucial determinant of the final ring structure of the zeolite.^33^ The mechanism of the formation of 3-ring and 4-ring configurations is similar to that observed in the simulations of linear structure formation, as elucidated in earlier computational studies.^27,49^Figure 5 displays snapshots of the calculated 4-ring reaction pathway in the presence of anion Cl^–^. During the first step, the active oxygen atom at one end of the linear tetramer binds to the Si atom at the other end, resulting in an intermediate ring. Subsequently, in the second step, the elimination of water yields the final product. Similar to previous studies,^27,49^ the leaving hydroxyl group is protonated through an external transfer mechanism, where it receives a proton from another water molecule in the surrounding. This is due to the specific arrangement of the hydrogen bond network around the silicate when the hydroxyl group moves apart from the 5-fold Si intermediate.

**Figure 6:**
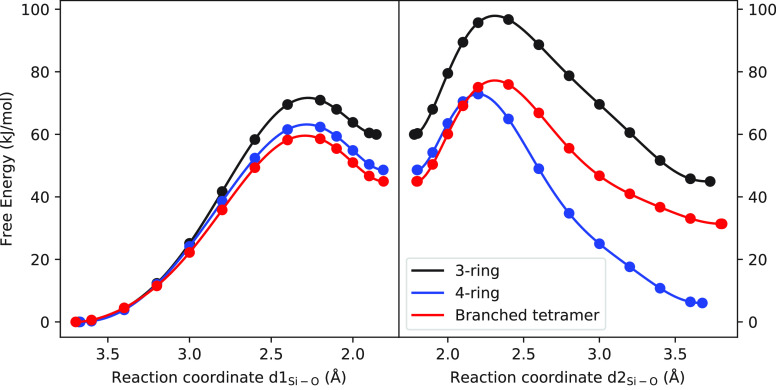
Calculated free energy profile of formation of ring and branched silicate oligomer as a function of the reaction coordinate.

Figure 6 illustrates the free energy profiles associated with the formation of 3-ring, 4-ring, and branched tetramer structures, with the respective numerical values tabulated in Table 3. The calculated results show that the formation of the 4-ring structure has the lowest free energy barrier (73 kJ/mol), which is only slightly lower than that of the branched-tetramer (77 kJ/mol). In contrast, the formation of the 3-ring structure is less favorable and requires a significantly higher free energy barrier (98 kJ/mol) compared to the 4-ring formation. Notably, this trend is opposite to the simulation results observed in the absence of the anion,^23^ where the 3-ring formation has a lower barrier than the 4-ring formation. These findings suggest that the presence of Cl^–^ affects the ring formation, resulting in a less favorable 3-ring formation pathway, with a higher free energy intermediate, transition state, and product structures compared to the 4-ring and branched tetramer pathways.

In agreement with previous theoretical studies,^24,28,54^ the present work observes positive reaction free energies, which is calculated as the free energy difference between the product and reactant state. This can be attributed to the generation of an extra water molecule during the reaction, which causes an entropically unfavorable rearrangement of the water structure. It is important to note that the overall zeolite synthesis process is thermodynamically favorable, as evidenced by previous experimental studies.^33^ In the current work, we focus on the first steps of the synthesis route. Accounting also for the subsequent steps will yield a thermodynamically favorable process. Also note that a forward reaction can occur by the excess amount of reactant while the production of products remains limited due to their continuous involvement in subsequent reactions. The results of free energy calculations presented in Table 3 demonstrate that, in the presence of Cl^–^, the 4-ring formation has the most stable product (6 kJ/mol), while the formation of 3-rings exhibits the highest reaction free energy (45 kJ/mol) as shown in Table 2. This indicates that 3-ring formation is thermodynamically unfavorable. Our findings suggest that the presence of Cl^–^ in the zeolite synthesis process suppresses the formation of 3-rings. This observation is consistent with experimental results that show the dominance of 4-ring and D4R structures in the final NaA zeolite structure.^32^

### Interaction between Cl^–^ and Silicate Oligomers

**Figure 7:**
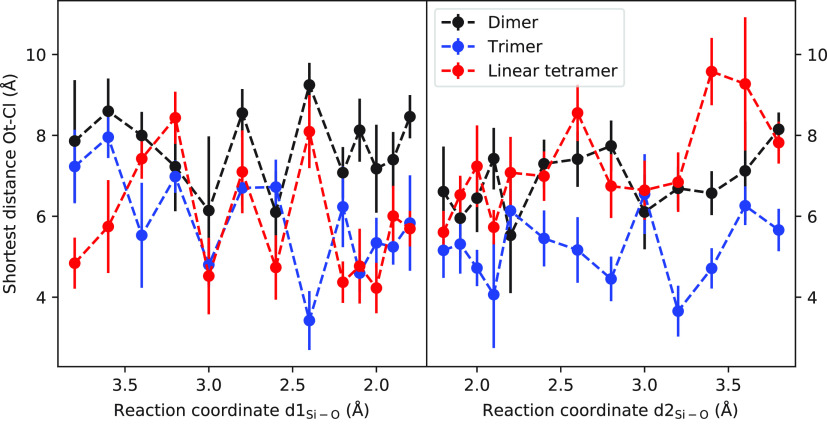
Shortest distance between Cl^–^ ion and oxygen of silicate as a function of reaction coordinate for linear silicate formation. The vertical bars indicate the variation (standard deviation) of the measured distances. Cl^–^ separates from the silicate in all cases.

In previous computational studies,^28,49^ it was observed that the height of the activation barrier varied with the relative position of the cation in the reacting species. In this study, we followed this analysis by investigating the relative position of the Cl^–^ ion with respect to the silicate species during the reaction. To do this, we measured distances between the Cl^–^ ion and the nearest oxygen atom of the silicate species and plotted the averages of these Ot-Cl distance distributions against the reaction coordinates, as shown in Figure 7. It is noteworthy that during the oligomerization reaction, ion Cl^–^ remains at a considerable distance from the silicate. This observation is consistent with the fact that both silicate and ion Cl^–^ are negatively charged. Hence, the electrostatic repulsion between the ions is substantial at short distances, leading to unfavorable interactions. This phenomenon can be attributed to the high charge density of the silicate and the significant size of the Cl^–^ ion. The presence of water molecules in the reaction medium can also contribute to the separation of the ions, as the hydration shell formed around the ions can further reduce the probability of a close contact between them. The distance between the Cl^–^ ion and the silicate also indicates that the influence of the Cl^–^ ion on the oligomerization reaction occurs mainly through its effects on the solvation shell of the silicate, rather than through direct interactions.

**Figure 8:**
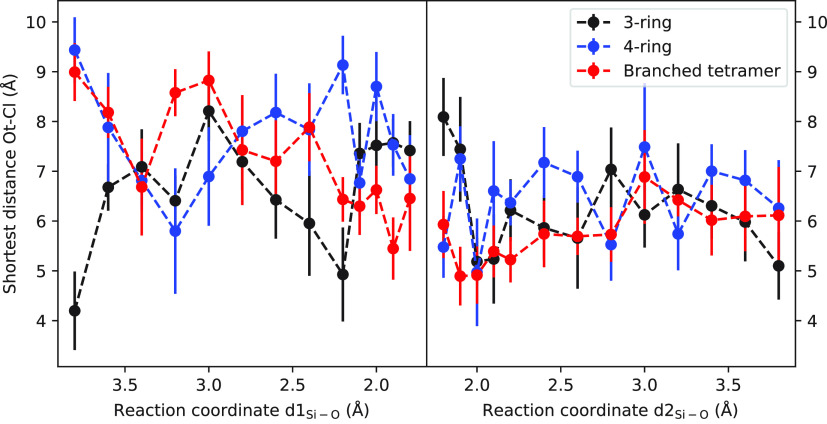
Shortest distance between oxygen of silicate and Cl^–^ ion as a function of reaction coordinate for ring and branched oligomer formation. The vertical bars indicate the variation (standard deviation) of the measured distances.

The shortest distance between the oxygen atom of the silicate and the ion Cl^–^ as a function of the reaction coordinate in the formation of ring and branched structures is depicted in Figure 8. Similar to the case of linear formation, throughout the entire reaction process of forming ring and branched structures, the ion Cl^–^ remains at considerable distance from the silicate. This observation is consistent with the repulsive nature of the electrostatic interactions between the negatively charged silicate and Cl^–^ ion, which becomes more significant at shorter distances. Hence, the ion Cl^–^ does not appear to have any direct interaction with the silicate during the formation of ring and branched structures.

## Conclusions

In this study, we investigated the effect of the anion Cl^–^ on the formation of silicate oligomers ranging from dimer to 4-ring using *ab initio* molecular dynamic simulations with explicit water molecules. Our results demonstrate that the presence of Cl^–^ leads to an increase in the free energy barriers of all reactions compared to the case without an ion. Specifically, the addition of either positively or negatively charged ions raises the activation energy barrier of the silicate condensation reaction. These findings are consistent with earlier computational studies of systems containing other cations.^27,49^

The observed increase in free energy barriers in the presence of Cl^–^ can be attributed to the disruption of the hydrogen bond network in the surrounding water molecules. This, in turn, leads to a decrease in the mobility of the water molecules and an increase in their entropic contribution to the overall reaction. Our results suggest that the presence of Cl^–^ has an effect on the silicate condensation reaction similar to that of other cations, highlighting the importance of considering the role of counterions in the synthesis of zeolites and other silicate materials.

Interestingly, our findings suggest that the formation of the 4-ring has the lowest free energy barrier, implying that it is kinetically favored over other oligomer structures. Notably, we observed that the formation of the 3-ring is the rate-limiting step in silicate growth in the presence of Cl^–^ due to its relatively high free energy barrier. The free energy barrier of the 4-ring is lower than that of the 3-ring by 25 kJ/mol. These observations suggest that the presence of Cl^–^ can significantly affect the kinetics of silicate growth with potential implications for the synthesis of zeolites and other silicate materials.

The results from AIMD simulations show that the formation of the 4-ring structure has a lowest free energy barrier and lowest reaction free energy compared to the formation of linear tetramer, branched tetramer, and 3-ring structures in the presence of Cl^–^. This suggests that the 4-ring structure is dominant in the synthesis of NaA Zeolite in the presence of Cl^–^, which is consistent with experimental observations where only the 4-ring structure was detected.^32^

In summary, our findings shed light on the role of Cl^–^ as a regulator of the thermodynamic and kinetic parameters in silicate oligomerization. The presence of Cl^–^ promotes the formation of 4-ring and larger oligomers by altering hydrogen bonding in the solvation shell. These results provide a foundation for future investigations into the formation of double ring structures in solution from a single ring as well as for larger scale kinetic Monte Carlo simulations. Such simulations, which can take into account the effects of silicate and Cl^–^ concentration, will provide a more detailed and comprehensive understanding of the underlying mechanisms and enable a more direct comparison with experimental observations.
